# An empirical study of the use of neuroscience in sentencing in New South Wales, Australia

**DOI:** 10.3389/fpsyg.2023.1228354

**Published:** 2023-08-22

**Authors:** Armin Alimardani

**Affiliations:** School of Law, University of Wollongong, Wollongong, NSW, Australia

**Keywords:** neurolaw, criminal law, neuroscience, biosciences, technology, criminal justice, sentencing

## Abstract

While neuroscience has been used in Australian courts for the past 40 years, no systematic empirical study has been conducted into how neuroscientific evidence is used in courts. This study provides a systematic review on how neuroscientific evidence is considered in sentencing decisions of New South Wales criminal courts. A comprehensive and systematic search was conducted on three databases. From this search, 331 relevant sentencing decisions before 2016 that discussed neuroscientific evidence were examined. The findings of this study suggest that neuroscientific evidence appeared to contribute to sentencing decisions in less than half of the cases examined; and in the majority of these, it supported a more lenient sentence.

## 1. Introduction

The increased academic interest in the intersection of neuroscience and law has given rise to many scholarly discussions and claims on the use of neuroscientific evidence in courts (e.g., see Hodgson, 2000; Greene and Cohen, 2004; Morse, 2006, 2011; Shen, 2010; Glannon, 2011). While neuroscientific evidence has been used for decades in courts across various jurisdictions, most studies on the use of neuroscience in courts have relied upon a limited number of cases involving neuroscientific evidence or mere theoretical conjecture (See, Vidalis and Gkotsi, 2012; Alimardani, 2018).

From 2015, a few studies from several jurisdictions, namely England and Wales, Canada, the United States, Slovenia and the Netherlands (hereafter ‘empirical neurolaw studies’), have conducted large-scale empirical research and presented analyses of the use and influence of neuroscientific evidence in criminal courts (see Catley and Claydon, 2015; de Kogel and Westgeest, 2015; Denno, 2015; Chandler, 2016; Gaudet and Marchant, 2016; Hafner, 2019). According to the analysis conducted in the present study, neuroscience has been used in Australian courts for the past 40 years. However, the previous studies in Australia have only either examined the potential application of neuroscience in courts in theory or have tended to draw upon a small sample of cases, providing only a qualitative assessment (see, Houston and Vierboom, 2012; Chau, 2015; Page, 2017; McCay and Kennett, 2019; McCay and Ryan, 2019). In one recent study in Australia, Alimardani and Chin (2019) briefly discussed various neurolaw claims in Australian courts. However, this study was also limited to a relatively small number of cases and provided only qualitative analysis (Alimardani and Chin, 2019).

In the absence of such empirical examination, neuroscience and law discourse has been limited to *theories* formed based on critics’ experiences from their interaction with the criminal justice system, speculation, and media reports of high-profile cases involving neuroscience (Farahany, 2016, 485). This is concerning because the results of the empirical neurolaw studies suggest some inconsistencies between how neuroscience is used in *practise* and how its use in courts is understood in *theory* (see, for example, Denno, 2015; Gaudet and Marchant, 2016).

To fill this gap in Australian literature, this study conducts the first systematic qualitative and quantitative analysis of the use of neuroscience in criminal courts. More specifically, this study examines whether and how neuroscientific evidence contributes to sentencing decisions. A comprehensive and systematic search was conducted on three databases. From this search, 331 sentencing decisions in New South Wales (NSW) before 2016 that discussed neuroscientific evidence were examined.

The empirical data from the results of this study reveal how different types of neuroscientific evidence are being considered in sentencing decisions, which sentencing factors are commonly associated with neuroscience, and whether neuroscientific evidence supports a harsher or a more lenient sentence. It will also address a recurring claim in neurolaw literature that neuroscience may have a double-edged sword effect: evidence of an impaired brain may suggest the offender is less culpable for the offence, simultaneously it may suggest the offender is dangerous and likely to reoffend.^1^ This study will also provide some practical insights for defence lawyers designing their strategies by revealing which specific aggravating and mitigating sentencing factors are often associated with neuroscience, and how frequently. Overall, the findings of this study will lead to a clearer understanding of the use of neuroscientific evidence in Australian courts (especially in New South Wales), and will formulate an empirical basis for neurolaw discussions.

Although this study focuses on New South Wales, this jurisdiction has considerable similarities with other Australian jurisdictions, and, therefore, could potentially represent the use of neuroscience in Australia generally.

This study does not claim that it can determine whether neuroscientific evidence actually increases or decreases a sentence or distinguish the impact of neuroscience on sentence from other forms of evidence.^2^ A challenge in examining the impact of neuroscience on sentencing is the difficulty in analysing and understanding how judges decide on the most appropriate sentence. While there are some general guiding principles for judges, the task of formulating an appropriate sentence is primarily a matter of judicial discretion, and it is not possible to determine, quantitatively, the extent to which a specific piece of evidence or relevant sentencing factor has influenced a sentencing decision. A judge may also discuss their sentencing remarks by referring to several pieces of evidence (ie, psychiatric and neuroscientific evidence) that report on the same issue (eg, the individual’s mental state), making it difficult to understand how the court has considered each piece of evidence in deciding the sentence, whether the sentence would have been different without a specific piece of evidence, or whether a specific piece of evidence was more influential than another.

## 2. Materials and methods

This systematic review was conducted in accordance with the Preferred Reporting Items of Systematic Reviews and Meta-Analyses (PRISMA) guidelines.

### 2.1. Defining search terms

In this study, similar to other empirical neurolaw studies, judgements in criminal cases were used as the source of data (de Kogel and Westgeest, 2015; Denno, 2015; Farahany, 2016). In order to find cases involving neuroscience, it is important to first define neuroscientific evidence. There is a lack of consensus in the neurolaw literature as to what constitutes neuroscientific evidence. Currently, there are two types of evidence that are the focus of neurolaw scholarly discussions: imaging evidence and non-imaging evidence. Imaging evidence is widely and conventionally known as neuroscientific evidence (see Catley and Claydon, 2015, 514), and includes computed tomography (“CT”), positron emission tomography (“PET”), magnetic resonance imaging (“MRI”), single-photon emission computed tomography (“SPECT”), functional magnetic resonance imaging (“fMRI”), magnetoencephalography (“MEG”) and electroencephalogram (“EEG”) scans.^3^ Non-imaging evidence refers to information derived from an individual’s medical history and tests conducted by medical professionals using measurements other than brain imaging techniques (eg, neuropsychological testing and psychiatric medical examination) that predict whether brain abnormalities exist and how this may be associated with their behaviour (Farahany, 2016). Unlike with imaging evidence, there are controversies around whether non-imaging evidence should be regarded as neuroscientific evidence.^4^

Similar to other empirical neurolaw studies, both imaging and non-imaging evidence were considered as neuroscientific evidence in this study,^5^ and search terms were chosen based on this definition. The results, however, were analysed once based on the narrow definition (imaging evidence only) and once based on the broad definition of neuroscience (including both imaging and non-imaging evidence). In this way, the results of this study may have utility for those who define neuroscientific evidence differently.

Some of the search terms used in this study to find relevant cases are: Ceretec Brain Perfusion Study, EEG, MRI, MMPI, qEEG, Diffusion Tensor Imaging (DTI), Rorschach Inkblot Test, PET, WAIS-R, SPECT, Magneto Encephalo Graphy (MEG), neuropsychological testing, neuropsychiatric testing, psychometric testing, brain, frontal lobe abnormality, Haemorrhage, brain injury, head injury, Ischaemia (or Ischemia), Cerebral Insult, neuroimaging, Hypoxia, BEAM. Since different brain imaging and tests in judgements are mentioned in different forms (eg MRI and Magnetic Resonance Imaging), all the different ways of referring to them were considered in the search.

### 2.2. Database search strategy

The selected search terms were used in three databases: CaseLaw,^6^ Australasian Legal Information Institute (“AustLII”)^7^ and the Australian Neurolaw Database (Neurolaw, n.d.)^8^ (which is a database that provides summaries of judgements involving neuroscience). The searches were confined to sentencing decisions in the courts of New South Wales, Australia, delivered on issues commenced before 31 December 2016. Five court tiers were targeted as they were most likely to hear criminal cases that discuss the mental state of the offender and, therefore, have a higher likelihood of neuroscientific evidence being raised: The High Court of Australia (only cases on appeal from NSW courts), the Court of Criminal Appeal, the Supreme Court, the District Court, and the Local Court.

### 2.3. Eligibility criteria and study selection process

All cases found in the databases were reviewed and those that were not relevant to the sentencing of the defendant were excluded, such as cases that concerned fitness to stand trial, where neuroscientific tools had been used to scan the body of the offender rather than the brain, civil law cases, duplicate cases generated due to the use of three databases, and where neuroscientific evidence was used to examine the victim or deceased.

**Figure 1:**
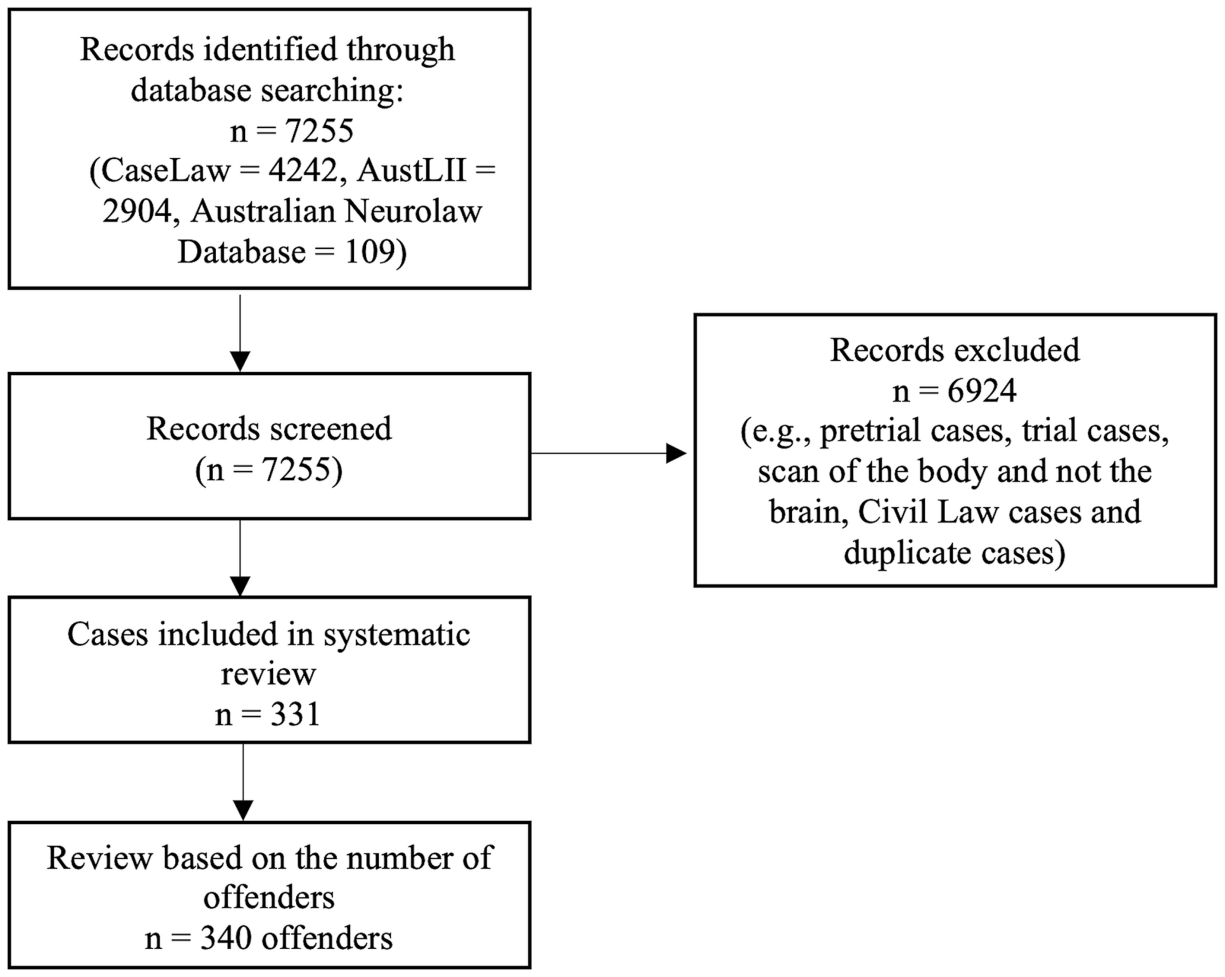
PRISMA summary of the study selection process.

The searches produced a total of 7,255 cases. Following excluding irrelevant and duplicated cases, 331 cases remained. During the case analysis, it became apparent that in five judgements there was more than one offender who was the subject of neuroscientific examination.^9^ As such, the analytical approach was changed from case-based to offender-based. That is, instead of considering the results by reference to 331 cases, this research analysed the 340 offenders in the sample. Nevertheless, to prevent any confusion, I will use terms “case” and “judgement” instead of ‘offender’ (ie, 340 cases/judgements). Figure 1 shows the PRISMA flowchart of this process. In 64 cases, both brain imaging and non-imaging evidence were introduced, and 276 cases contained no imaging evidence (Table 1).

**Table 1:** Number of cases based on evidence type.

Description	At least one imaging evidence	Contains no imaging evidence	All cases
Number of cases	64	276	340

### 2.4. Data extraction and coding

After the selection process, all the cases were coded, ie, each case was analysed against more than a hundred key factors, such as the type of neuroscientific evidence discussed, sentencing elements associated with neuroscience, the year of judgement and court tier. To ensure a consistent analysis, the Qualtrics platform was used. At first, 10 cases were selected and coded to assess the appropriateness of the key factors and whether they reflect on all the relevant aspects of the study. Many areas that required to revise the key factors were identified and therefore, piloting the cases continued for another 50 cases until there was no further area of improvement. All 60 cases were coded again against the final version of key factors.

### 2.5. Study quality and the risk of bias assessment

One of the main limitations of using criminal cases as the source of data in this research is that not all judgements are transcribed and uploaded to electronic databases, and the collection of neurolaw cases that are accessible online only represents a proportion of the actual number of cases.^10^ As it is impossible to determine the actual number of cases where neuroscience has been introduced in courts if they have not all been published in these electronic databases, this study cannot provide a definitive conclusion as to how neuroscientific evidence is being considered in NSW courts. Nonetheless, the collected cases in this study are capable of generating a useful representation of the courts’ general position on the influence of neuroscientific evidence on sentencing. Further, it is these published cases that are cited in future judgements in a common law system and, therefore, the cases analysed may inform future precedents and court procedures concerning the use of neuroscientific evidence in sentencing (see Chandler, 2016).

Relying on criminal judgements as a source of data, this research is limited to what judges include in their sentencing judgements (Al-Alosi, 2016). Some courts may not include all the details from a neurologist report or may simply summarise matters that are important to the objectives of this study. More importantly, the researchers are blind to many of the factors that may influence a court’s decision such as the media (see White et al., 2012) and conscious and unconscious biases (see Spohn, 2000; Steffensmeier and Demuth, 2000, 2006; Leiber and Fox, 2005; Franklin and Fearn, 2008; Doerner and Demuth, 2010; Goodman-Delahunty and Sporer, 2010). Further, similar to many other forms of evidence, the interpretation of neuroscientific evidence relies on the subjective analysis of expert witnesses. Therefore, the implication of neuroscientific evidence in a judgement may differ from one expert witness to another (Dumit, 1999). This variability in interpretation becomes even more complex when considering the qualifications of those interpreting the evidence. According to some scholars, certain expert witnesses who interpret neuroscientific evidence lack the requisite expertise and qualifications, rendering their reports and testimonies unreliable or lacking in credibility (see Bremner, 2005; Amen and Flaherty, 2006; Moriarty and Langleben, 2017). It is unclear from this study whether courts are concerned with or possess the knowledge of the required qualifications to report on different forms of neuroscientific evidence and whether that knowledge would impact the outcome of the cases.

Another limitation of this study is associated with the subjective analysis of content in cases. Two individuals may make different interpretations of a matter in a judgement and accordingly make different conclusions. The case analyses in this study do not benefit from the inter-rater reliability approach^11^ — that is, where two researchers separately analyse a judgement and then compare their coding results to avoid the impact of subjective interpretation on the text (see, eg, Farahany, 2016). In some cases, due to the complexity of judgements or where the court was silent or unclear about the relevance of neuroscience to sentencing, a determinative decision could not be reached as to whether neuroscience was relevant to particular aggravating and mitigating factors. In such circumstances, those sections of the judgement were coded as “unclear.”

## 3. Results and discussion

### 3.1. Sentencing factors associated with neuroscience

Before discussing the outcome of the systematic review of cases and different ways in which neuroscience contributes to sentencing decisions in practise in the next Part (qualitative analysis), this Part will provide a summary of sentencing factors that are potentially associated with neuroscience and how they serve to increase or decrease the allocated punishment. This review is specifically useful for those who are not familiar with the NSW criminal justice system.

Factors that may mitigate or aggravate a sentence are recognised in legislation – section 3A (Purposes of Sentencing) and section 21A (Aggravating, Mitigating and Other Factors in Sentencing) of the *Crimes (Sentencing Procedure) Act* 1999 (NSW) (“*Sentencing Act*”) – and the common law. It should be noted that the aims of sentencing do not operate as mitigating or aggravating factors in and of themselves. Nonetheless, to make the discussions more concise, I will refer to them as mitigating and aggravating factors depending on the circumstance of the case, eg where I refer to specific deterrence as a mitigating factor, this means that the court has given it lower or no weight and therefore supports a shorter sentence.

*Moral culpability* is a sentencing consideration that refers to the degree to which an offender is blameworthy. A brain condition that causally contributes to the commission of an offence by reducing the offender’s capacity ‘to understand the wrongfulness of [their] action, or to make reasonable judgements, or to control [their] faculties and emotions’, may reduce the offender’s *moral culpability* and the severity of punishment (*R v Israil* [2002] NSWCCA 255, [23]).^12^

The offender’s *prospects of rehabilitation* is another sentencing consideration^13^ and is closely associated with the *protection of society* from the offender during the period they are kept in prison.^14^ A brain impairment that is causally connected to offending may suggest a high risk of recidivism, but if the court concludes that this condition is treatable and the offender has good prospects of rehabilitation, it may result in a consideration that the offender is unlikely to reoffend.^15^ The interplay between these two sentencing factors is an important one when it comes to neuroscientific evidence. According to one Australian report, it appears there is a concerning attitude held by the courts towards rehabilitation of offenders with an acquired brain injury, that nothing works for them (ie, therapeutic nihilism), while there are many studies that suggest otherwise (Rushworth, 2011, 27).

*Deterrence* aims to use punishment or the threat of punishment to dissuade the offender from reoffending (specific deterrence) and the public at large from engaging in similar crimes (general deterrence) (*Sentencing Act* s 3A(b)). An offender with a brain condition is not an appropriate vehicle with which to deter the community and therefore, the court may attach lower or no weight to general deterrence (see *R v Engert* (1995) 84 A Crim R 67, 71 (Gleeson CJ), discussing *R v Scognamiglio* (1991) 56 A Crim R 81, 86). If an offender commits a crime as a result of their brain abnormality, but they have good prospects of rehabilitation and the risk of recidivism is slight, the court may give lower or no weight to the importance of specific deterrence in sentencing (see Odgers, 2015). On the other hand, if ‘it is not shown to be likely that the offender will accept the need for treatment in the future’, the court may give more consideration to the importance of specific deterrence and impose a harsher punishment (Odgers, 2015, 277, quoting *Clay v The Queen* [2007] NSWCCA 106, [25]–[6]).

In circumstances where neuroscientific evidence suggests that the offender’s brain condition makes *imprisonment more onerous* (ie, custodial hardship) than the average prisoner (Freckelton, 2019), or where the custodial sentence would have a *significant impact on the offender’s health*, the court may mitigate the punishment (*R v Smith* (1987) 44 SASR 587, 589 (King CJ)). The court may also mitigate the sentence if the brain impairment causes an offender to *not fully appreciate the consequences of their conduct* (*Sentencing Act* s 21A(3)(j)).

Overall, it appears that neuroscience may potentially aggravate the sentence by way of only two of the sentencing factors — specific deterrence and protection of society — and the rest, when associated with neuroscience, tend to mitigate the sentence.

A deciding factor for defence lawyers as to whether to adduce neuroscientific evidence, in circumstances where there are one or more factors that support a harsher punishment and simultaneously one or more factors that support a more lenient sentence (ie, the double-edged sword), is which side potentially outweighs the other.

While it is not possible to qualitatively determine how much one sentencing factor may influence the sentence, Stephen Odgers, in discussing cases where the evidence of mental abnormalities may support both a shorter and a longer sentence, explains that: ‘more commonly, a mental abnormality will result in a more lenient sentence than would otherwise have been imposed’ (Odgers, 2015, 278). More importantly, the High Court in *Veen v The Queen [No 2]* (1988) 164 CLR 465 (“*Veen No 2*”), with respect to cases where a mental condition results in consideration of countervailing effects of moral culpability and protection of society — one tending towards a more lenient sentence and the other tending towards a more harsh sentence — stated that ‘[t]hese effects may balance out, but consideration of the danger to society cannot lead to the imposition of a more severe penalty than would have been imposed if the offender had not been suffering from a mental abnormality’ (at 465, 477, Mason CJ, Brennan, Dawson and Toohey JJ).

These conclusions appear to be associated with the consideration of objective and subjective sentencing factors. In determining the sentence, the punishment should not go beyond or below what is proportionate to the objective seriousness of the offence (*Sentencing Act* s 3A(a))^16^ — those factors that are associated with the circumstances of the offence and not the offender. However, if the offender has a mental condition, which is a subjective factor (those that are associated with the circumstances of the offender and not the offence), and it is causally connected to the commission of the offence, then it would be a matter relevant to the assessment of the objective seriousness of the crime (*Director of Public Prosecutions (Cth) v De La Rosa* (2010) 79 NSWLR 1, 43 [177] (McClellan CJ at CL), cited in *Tepania v The Queen* [2018] NSWCCA 247, [118] (“*Tepania*”)).^17^ In this regard, the court in *Tepania* at [112] explained that,

In sentencing for an offence … a court should make an assessment of the objective gravity of the offence applying general law principles, so that all factors which bear upon the seriousness of the offence should be taken into account … Regard may be had to factors personal to the offender that are causally connected with or materially contributed to the commission of the offences, including (if it be the case) a mental disorder or mental impairment.

As such, factors that may explain why an offence was committed, eg, a mental condition that reduces the offender’s capacity to make reasonable judgements, by way of reducing the significance of moral culpability,^18^ may in turn lead to the imposition of a sentence that would otherwise be below the lower limit of the sentence, proportionate to the objective seriousness of the offence (see *Elturk v The Queen* 239 A Crim R 584, 591–2 [33]–[5]; Howard and Westmore, 2010, 530–34).^19^

However, courts consider subjective factors — ie, factors that are not associated with the circumstances of the offence such as the offender’s prospects of rehabilitation and risk of recidivism — to determine the most appropriate punishment within the range of a proportionate sentence and cannot *extend* the punishment beyond what would have been proportionate had the mental illness not existed.^20^

Concerning the consideration of the protection of society in sentencing, in *Veen No 2* it was held that:

The principal for proportionality is now firmly established in this country … a sentence should not be increased beyond what is proportionate to the crime in order merely to extend the period of protection of society from the risk of recidivism on the part of the offender (at [472], Mason CJ, Brennan, Dawson and Toohey JJ) (citations omitted).

Overall, an important distinction between objective and subjective factors in this study is that objective factors can alter the proportionate punishment, while subjective factors can only influence the sentence within the range of proportionate punishments, meaning objective factors are potentially more influential than subjective factors.

## 4. A qualitative analysis of neuroscientific evidence in sentencing

The section provides a qualitative analysis of several representative cases that demonstrate four different ways in which neuroscience is relevant to sentencing. This analysis provides particularly meaningful context for the next section, which provides an analysis of the quantitative data — the number of judgements where neuroscience has supported a more lenient or more harsh punishment.

### 4.1. Neuroscience pointing towards a shorter sentence

In *R v Lea-Caton* [2007] NSWSC 1294, the offender pleaded guilty to four offences: the murder of two people (maximum penalty of life imprisonment) and two counts of a specially aggravated form of kidnapping (maximum penalty of 25 years imprisonment). On the day of the incident, the offender kidnapped the victims with two co-offenders. The offender was responsible for guarding the victims but was not engaged in attacking and tying them. He was present at the scene when the murder was committed by the co-offenders but did not physically contribute to killing the victims. He also assisted the co-offenders in moving their bodies to a remote area to burn them. Shortly after the crime, the offender surrendered himself.

According to the offender’s medical history, he was involved in a serious motor vehicle accident where he ‘sustained severe injuries including a depressed fracture of the left frontal skull’. His sister noted that the accident changed his personality and mood, and it was difficult for him to make decisions when he was under pressure. He descended into a cycle of depression and attempted to commit suicide. Other acts that resulted from the apparent personality changes included frequent offences such as break and enter with intent to steal, drug crimes and dangerous driving. The information in the judgement does not suggest that he had been convicted of any other offences before the motor vehicle accident.

Based on an MRI scan of the offender’s brain, a clinical psychologist reported that the motor vehicle accident had caused ‘frontal lobe syndrome’—an impairment in the frontal lobe which may result in behavioural problems such as impulse control disorders, violent behaviour and impaired judgement (Yang and Raine, 2009). The expert witness also explained that the offender’s psychological assessment was consistent with the claim that the offender had issues with making decisions when he was under pressure. The Court accepted that the offender’s frontal lobe condition was a matter which “*somewhat* diminished [his] capacity … to have extricated himself from the situation in which he found himself on that fateful day” (at [45], emphasis added). Subsequently, the Court, in determining the objective seriousness of the crime, considered that the offender’s “psychological condition compromised his capacity to react appropriately to the circumstances in which he found himself” (at [59]) and ordered 22 years of imprisonment with a non-parole period of 16 years and 6 months.

This judgement is an example of a scenario where, due to the reduced capacity of the offender as a result of their brain injury — shown in neuroscientific evidence (MRI scan) along with other evidence (psychological evaluation and medical records) — the offender’s sentence was reduced by way of mitigating the significance of the objective seriousness of the crime. Neuroscience was not the sole factor considered in determining the offender’s mental condition; a psychological assessment and behavioural evidence provided by his sister also contributed to determining his diminished capacity. This combination of evidence demonstrates the difficulty that arises when distinguishing the influence of neuroscience from other sources in sentencing decisions.

In *Carroll v The Queen* [2012] NSWCCA 118 that involved no brain imaging evidence, the offender, following a fight, slashed his victim’s forearm and face with a knife. A few weeks after the incident he confessed to his crime. The offender suffered from loss of consciousness and mild amnesia as a result of two head injuries sustained in 1999 and 2002. He further experienced seizures and in the majority of episodes he was violent in the post-ictal period (ie, the 5–10 min after a seizure). Further, the seizure medications he was taking induced serious side effects that included worsening his mood and hindering his temper control. Nonetheless, a psychiatrist’s report indicated that his seizures were not directly connected with the crime. Instead, the expert witness suggested that the offender ‘probably suffered frontal lobe damage and that this would have contributed to a diminished capacity for self-control and increased his vulnerability to acting in an impulsive and aggressive manner when angered’ (at [30]).

The court did not refer to the possible causal contribution of the offender’s frontal lobe damage to the offence and after consideration of his guilty plea, delivered a sentence of 8 years and 3 months imprisonment with a non-parole period of 6 years.

The offender appealed against the sentence on several grounds, among them being the sentencing court’s failure to consider their mental condition. The Court of Criminal Appeal stated that it appeared that the sentencing court did not consider the contribution of cognitive issues to the offence and that the court should have considered the applicant’s brain injuries, seizures, and the side effects of his medication. The Court of Criminal Appeal noted that

significant weight must be given when determining a sentence to the impact that the applicant’s head injuries have had on his behaviour, including the fact that he has poor temper control … it seems obvious, and I would conclude, that poor temper control played a significant role in the events that occurred (at [102]–[03], Garling J).

The Court explained that due to the offender’s head injuries, less weight should be attached to his moral culpability. Following consideration of other sentencing factors, the Court unanimously imposed a non-parole period of 4 years with a remaining balance of 2 years.

In these two cases, and generally in many other cases in this study and the neurolaw literature, the discussions are mainly around how a brain abnormality contributed to the offence. However, even if a brain abnormality had not affected the offender’s behaviour, or if it did not exist at the time of offending (eg, it occurs after the offence in prison), neuroscientific evidence may still be relevant to sentencing considerations. For example, in *R v Clay, Lonsdale and JM* [2006] NSWSC 1220, three offenders were involved in a physical conflict that resulted in the victim’s death. Two of the offenders pleaded guilty to manslaughter and affray and the other offender, Lonsdale, pleaded guilty to manslaughter (Manslaughter has a maximum penalty 25 years). With respect to Lonsdale’s mental condition, a psychiatrist reported that he suffered from a head injury in football and that he was diagnosed with a schizoaffective disorder. An EEG examination indicated the presence of brain abnormalities and a SPECT scan indicated ‘abnormal perfusion in areas of the brain consistent with the presence of a psychotic episode’ (at [38]). However, CT and MRI brain scans of his brain showed no abnormalities.

Lonsdale’s medical history also indicated that 4 years prior to the offence, he was admitted to the hospital seemingly due to a typical drug-induced psychosis. Nonetheless, since his recovery and at the time of the offence, there were no recurring drug-induced psychoses or “symptoms of schizoaffective disorder.”

The defence, by referring to the offender’s psychotic illness and his brain abnormalities, requested a more lenient sentence. The court referred to different ways that mental illness may affect the sentence, including where it contributes to offending the offender’s moral culpability may be reduced, adjusting the significance of general deterrence, and that the sentence may weigh more heavily on him (ie, custodial hardship). However, the Court noted that the offender was sufficiently aware of his acts and that there was no causal relationship between the crime and the mental condition. Nonetheless, the Court, given other relevant mitigating factors made ‘some modest adjustment of the otherwise appropriate sentence’ and added that ‘It is appropriate to do so even though there is no direct evidence of a causal relationship between his condition and the commission of the offence’ ([65] (citation omitted)).

These three cases show how neuroscience may support a more lenient sentence regardless of whether the brain damage is causally connected to the criminal behaviour. In the following Part, I will move on to discuss cases where neuroscience tends towards a harsher punishment.

### 4.2. Neuroscience pointing towards a longer sentence

There are only two aggravating factors that are potentially associated with neuroscience: protection of society from the offender and specific deterrence. In cases where neuroscience tends towards a harsher punishment, protection of society is often the relevant sentencing factor associated with neuroscience, whereas consideration of specific deterrence to prevent further offending is only occasionally a relevant sentencing factor.

*Ngati v The Queen* [2014] NSWCCA 125 is the only case in the dataset of this study in which neuroscience is only associated with the aggravation of the sentence but not with any of the mitigating sentencing factors. In this case, the applicant was sentenced to 12 years imprisonment for two counts of armed robbery (maximum penalty of 20 years) which involved serious violence against the victims. Further, in association with each armed robbery, there were two additional charges of detaining a person for advantage in company and robbery in company — section 32(1) of *Sentencing Act 1900* (NSW) outlines that under Form 1 procedure, ie, during the sentencing procedure, the court may consider further offences which the offender has been charged with, but not convicted and the offender “wants the court to take into account when dealing with the offender for the principal offence” (*Sentencing Act 1900* (NSW) s 32(1)).

The offender had a significant criminal history, including offences such as robbery and arson. At the age of 17, he had suffered from substance abuse and the motive for his most recent offence was to supply his addiction. It was reported that his substance abuse was accompanied by mental health issues. A psychologist, based on the results of psychometric testing, reported that his IQ score ranged between 52 and 66 (ie, severe impairment in intellectual functioning). A psychiatrist, after examining the applicant, also concluded that he suffered from an impairment in intelligence and cognitive ability. Further, the applicant suffered from a head injury when he was 7, but none of the expert witnesses could conclude whether his head injury had a considerable influence on his offending behaviour.

The applicant appealed against the severity of the sentence. He argued that the sentencing judge did not provide due consideration to the influence of the applicant’s impaired intelligence on his offending behaviour and that the sentence should have otherwise been more lenient.

However, based on the fact that the crime had involved some element of planning and therefore was not an impulsive act, and that he had been completely aware that his behaviour was wrong, both the sentencing court and the appeal court concluded that there was no causal relationship between his impaired intellectual capacity and his criminal conduct.

Counsel for the applicant, however, argued that there was a causal relationship between the applicant’s mental condition and his criminal behaviour. Referring to the psychometric examination and the report provided by the psychologist, counsel noted that ‘[t]he likelihood that [the applicant] may return to substance use and related offending in times of stress may be aggravated by below-average intelligence, which can be associated with decreased control of [offending] behaviours and capacities for consequential reasoning’ (at [35]). However, the appellate court adopted a different interpretation of the expert’s report, explaining that this part of the report was referring to the applicant’s risk of recidivism rather than suggesting that his mental condition had contributed to the criminal conduct. Based on the provided evidence, the sentencing court concluded that the applicant had poor prospects of rehabilitation and that there was a considerable likelihood of recidivism. Accordingly, the results of the psychometric examination, showing a considerable impairment in intellectual functioning, only pointed towards a harsher punishment.

### 4.3. Neuroscience cutting both ways: supporting a more harsh and a more lenient sentence simultaneously

The previous two Parts discussed how neuroscience might tend towards either mitigating or aggravating the sentence. The following will provide some cases that illustrate how neuroscience may contribute towards both mitigating and aggravating the sentence simultaneously.

One example involves the sentencing decision in *R v McCann* [2012] NSWSC 1462. The offender was found guilty of manslaughter. During a struggle between the offender and the deceased, the offender wrapped a nylon rope around the victim’s throat and strangled him to death. Several expert witnesses evaluated the offender’s mental condition. A clinical psychologist reported that he had a cognitive impairment that was consistent with frontal lobe damage. This type of injury potentially causes problems in the sufferer with regards to planning, mental flexibility, social judgement and in determining appropriate behavioural responses. A neuropsychologist’s assessment of McCann’s CT and MRI scans revealed the existence of focal abnormalities in the frontal lobes and cerebral atrophy. The expert also reported that the offender had cognitive problems including issues with learning and remembering complex new information.

Three psychiatrists, after reviewing the neuropsychologist’s reports (including those on McCann’s brain images), made similar assessments that at the time of the offence, McCann had ‘atrophy of the frontal lobes and general brain shrinkage’ at the time of the offence (at [22]) that led to cognitive impairments (ie, an impairment of the brain’s executive function) and increased the possibility that he would exhibit poor decision-making, misunderstand events and act impulsively.

On the one hand, the Court acknowledged that McCann’s mental condition led them to consider that general deterrence and retribution were less important. On the other hand, the court added that, ‘the irreversible condition of the frontal lobes’ increases the offender’s risk of recidivism (at [29]). Although the psychiatrists had conflicting opinions about his risk of recidivism, the Court concluded that the offender carried a high risk of showing violent behaviour once released and thus posed a particular risk to society. Accordingly, in this case, neuroscientific evidence simultaneously pointed towards mitigating and aggravating the sentence (For a similar discussion on this case, see Alimardani and Chin, 2019).

*Veen v The Queen [No 1]* (1979) 143 CLR 458 (‘*Veen No 1*’) and *Veen No 2* are two further examples where neuroscientific evidence pointed towards both mitigating and aggravating the sentence simultaneously.

In the trial court in both cases, Veen was found guilty of manslaughter while acting under a substantial abnormality of the mind arising from his brain damage. The Courts in both cases found that Veen’s brain condition contributed to his offending and consequently that he deserved a more lenient punishment. However, the courts noted that Veen’s brain condition also meant that once he was released from prison, he would be a grave danger to society.

The double-edged sword effect of neuroscience is more clearly discussed in *Veen No 2*, where the Court, concerning Veen’s brain issues, explained that:

[the purposes of sentencing] are guideposts to the appropriate sentence but sometimes they point in different directions. And so a mental abnormality which makes an offender a danger to society when he is at large but which diminishes his moral culpability for a particular crime is a factor which has two countervailing effects: one which tends towards a longer custodial sentence, the other towards a shorter (at 476–77, Mason CJ, Brennan, Dawson and Toohey JJ).

Although the Court does not refer to any type of brain imaging evidence in either of these cases, this discussion of the medical examination of Veen’s brain condition shows that, from over three decades ago, the courts have recognised neuroscience as a type of evidence that may support a more lenient sentence and a harsher punishment simultaneously. Also, as the *Veen* cases appear to be two of the earliest instances in which neuroscience has been relied upon by an Australian court, this may suggest that over the 30 years since *Veen*, neuroscientific evidence has continued to have both a mitigating and aggravating effect in many other judgements. However, as I will discuss in the fourth Part of this article (the quantitative analysis of this study), the results show the opposite.

### 4.4. Neuroscientific evidence suggesting a reduced risk of re-offending

Other than the three ways in which I have discussed how neuroscience is associated with sentencing — as mitigating, aggravating or both — neuroscience evidence has been considered in another way in the sentencing procedure: the court may consider the brain impairment as a factor that *reduces* or *eliminates* the risk of re-offending. In *R v Goodridge [No 2]* [2012] NSWSC 1180, the offender forced his arm in the victim’s vagina and rectum causing heavy bleeding and death. He was found guilty of murder. The offender had suffered from two head injuries; one when he was 3 and the other in 2010 from an assault in prison. He had started drinking alcohol and using drugs from an early age. Four expert witnesses provided reports of the offender’s mental assessment.

A psychiatrist and a psychologist suggested that the existence of brain damage was due to his long-term consumption of alcohol and previous head injuries and found that the offender had poor judgement and a low baseline IQ. Another psychiatrist, by examining his MRI brain scans, suggested that he had ‘cerebral atrophy and lesions in the white matter consistent with vascular disease’ (at [31]). The third psychiatrist, following a cognitive test, confirmed his diagnosis of dementia and that Goodridge could not remember committing the crime, his trial, nor why he was in prison. The psychiatrist further explained that if his cognitive decline continues, he would not live more than 6 to 7 years.

After considering the expert witnesses’ reports, the sentencing court noted that his history of alcohol abuse and his brain damage indicated that he was prone to binge drinking which tended to make him act aggressively. The offender’s judgement was impaired and therefore his moral culpability and the need for general and specific deterrence were reduced. With respect to future dangerousness, the court found that due to his deteriorating mental and physical health condition, he ‘no longer present[ed] any danger to the community’ and would likely pass before completing his sentence (at [46], [47]).^21^

As such, the possibility of increasing the offender’s sentence based on the likelihood of future offending was eliminated due to his compromised physical and mental state (ie, dementia), and although his brain impairment was causally associated with the conduct of a violent murder, neuroscientific evidence only pointed toward a more lenient sentence.

In *R v Caleb James O’Connor* [2013] NSWDC 272, the offender sustained a brain injury *after* the crime. The offender was found guilty on three counts: two attempts to choke a person with the intention of committing an indictable offence (ie, intimidate the victim) and an offence of sexual intercourse without consent. About a week after the trial, the offender suffered from a major brain injury as a result of an assault in custody. Expert witnesses found that several cognitive issues resulted from this injury including amnesia (eg, he had problems remembering the crime), anxiety and changes in his personality.

The Court found that due to his brain injury, general and specific deterrence were less significant and that further custody would interrupt his rehabilitation process: ‘[i]t must be recognised that the longer he spends in custody the greater is the risk to that rehabilitation’ (at [100]). Despite the offender’s criminal record and the presence of dynamic risk factors such as problems with his sexual-and self-regulation, an antisocial personality, and negative emotional responses, the Court found that due to his amnesia and the severity of the injuries, the significance attached to the protection of society had now been reduced compared to before the offender had suffered the assault.

In this case, unlike in *Goodridge*, the brain impairment was not a contributory factor to the original criminal behaviour. However, it still tended toward mitigating the sentence by way of general and specific deterrence. Neuroscientific evidence also altered the consideration of other factors that supported the risk of future offending.

Now that the different ways in which neuroscience may tend toward mitigating and aggravating the sentence have been discussed, the next Part will move on to discuss the results of the quantitative analysis.

## 5. A quantitative analysis of neuroscientific evidence in sentencing

**Figure 2:**
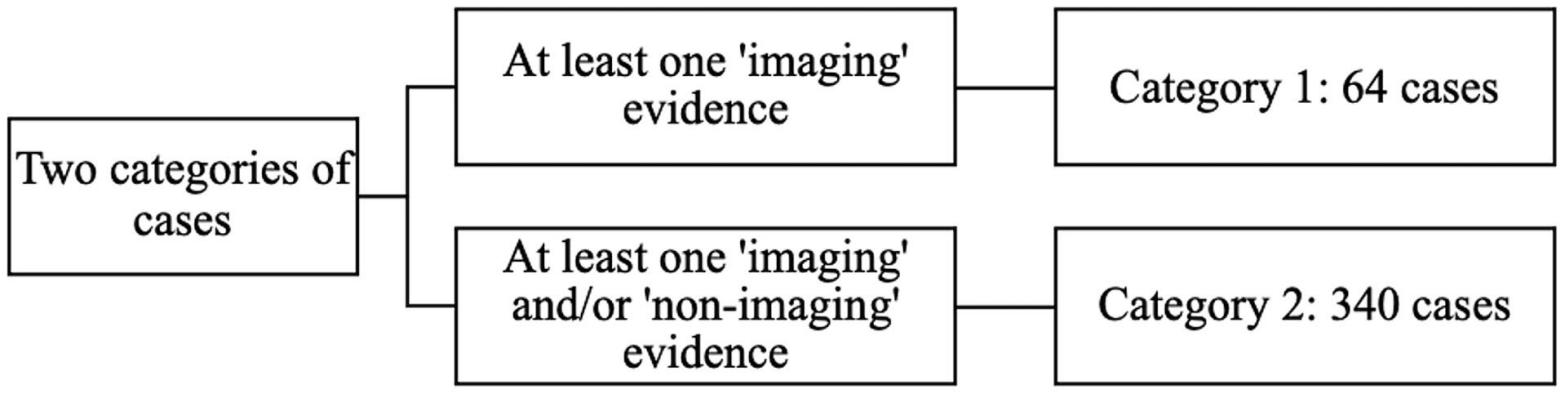
Two categories of cases.

The methodology Part discussed that there are some arguments and uncertainties over the definition of neuroscientific evidence. Neuroscientific evidence is divided into two types for the purposes of this study and to ensure that the results of this study have utility for different scholars who may define neuroscientific evidence differently. These two types are: imaging tools and non-imaging evidence. Criminal cases were placed into two categories based on these two types of neuroscientific evidence and the results of this study are presented based on these two categories. Category 1 contains cases where at least one type of ‘imaging’ evidence was introduced to the court (64 cases). Category 2 includes cases in which at least a form of either imaging evidence or non-imaging evidence, or both types were presented to the court (340 cases). Accordingly, Category 1 would suit scholars who define neuroscientific evidence narrowly, and Category 2 would suit scholars who define neuroscientific evidence more broadly to include both imaging and non-imaging evidence (Figure 2).

It is of note that while cases in Category 1 include instances of imaging evidence, often non-imaging evidence was also presented to the court. For instance, where an expert witness reports that the MRI scan shows brain abnormality, and that the offender’s behaviour is also consistent with an individual with some form of brain impairment. Despite this, due to methodological difficulties and uncertainties, distinguishing the influence of each type of evidence on the sentence presented to the court was not possible in this study (for a similar discussion, see Denno, 2011, 975). It is noteworthy that when analysing these cases, it was specifically examined whether the court found any association between the result of imaging evidence and sentencing. In more than half of the cases in Category 1, the imaging evidence suggested no brain abnormality or the results were unclear. Overall, the analysis and discussions in Category 1 are limited to 26 cases (out of 64) where the imaging evidence suggested some form of abnormality and the court considered this evidence in their sentencing decision.

Table 2, for both categories of cases, lists six rows of data: the first three rows show where neuroscience tends to only aggravate the sentence, only mitigate the sentence, and where it tends toward both aggravating and mitigating the sentence. The next row lists cases where the contribution of neuroscience to sentencing is unclear or where neuroscience does not contribute to the sentence — ie, neither mitigate, nor aggravate the sentence. To assist in understanding the proportion of cases that tended toward aggravating, mitigating or both, the last two rows represent cases where neuroscience contributed to the sentence in general (either aggravate, mitigate or both), and the total number of cases in each category.

**Table 2:** Comparison of the impact of two categories of evidence on sentence by the number of cases.

Description	Category 1 At least one “imaging” evidence	Category 2 At least one “imaging” and/or “non-imaging” evidence (all cases)
Only aggravating	0	1
Only mitigating	23	128
Both aggravating and mitigating	3	18
Does not contribute to the sentence or unclear	38	193
Contributed to sentence	26	147
Total cases	64	340

As can be seen in Table 2, in Category 1 (64 cases) — ie, where there is at least one imaging evidence — there are no cases where neuroscience contributed towards aggravating the sentence, compared to 23 cases in which neuroscience contributed toward a more lenient sentence. Further, there are only three cases in which neuroscience contributed towards both aggravating and mitigating the sentence simultaneously. Similarly, in Category 2 (340 cases) — ie, all cases — there is a significant difference between the number of cases where neuroscientific evidence supported a more lenient sentence (128 cases), against cases where neuroscientific evidence tended to aggravate the sentence (1 case). There are 18 cases in which neuroscience contributed to both aggravating and mitigating the sentence.

**Figure 3:**
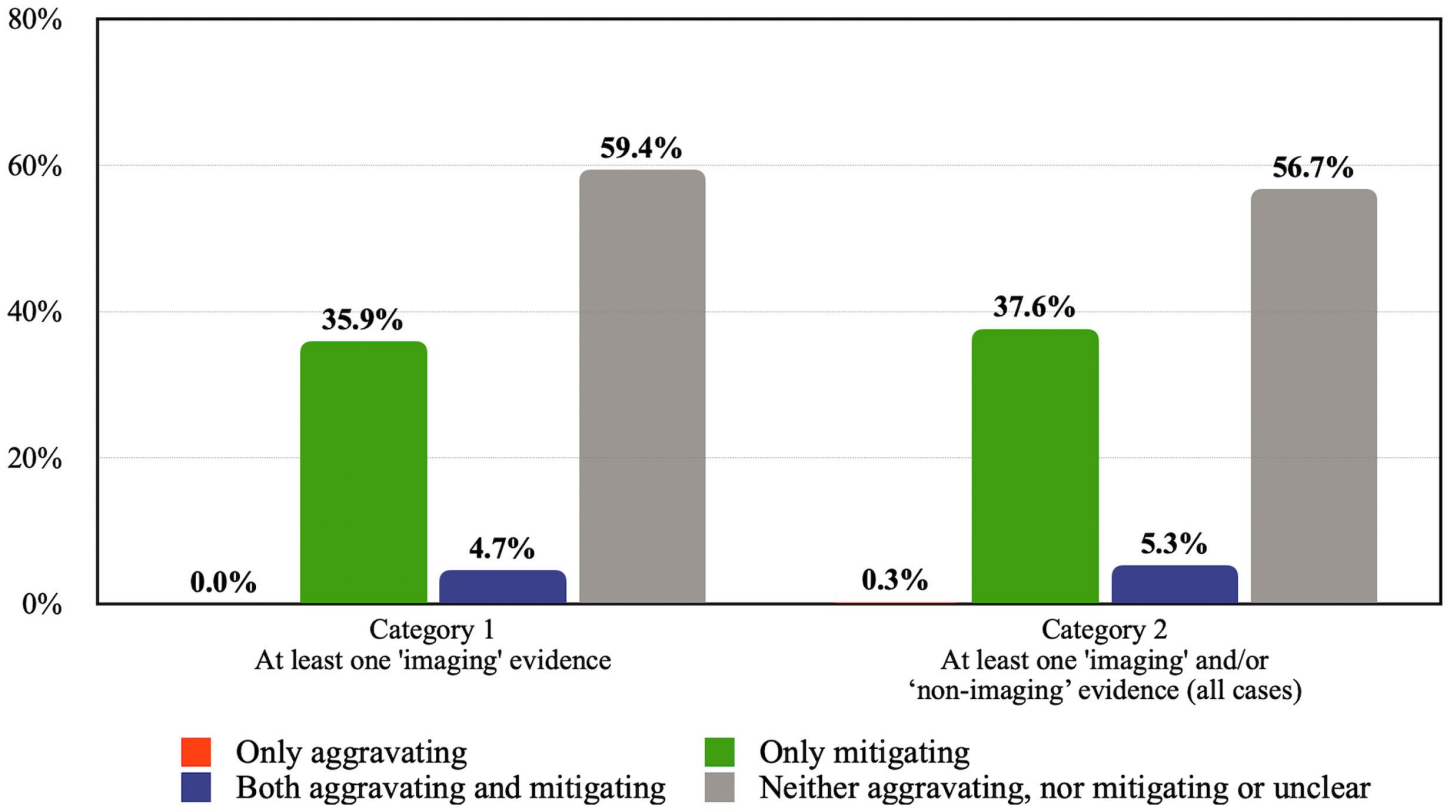
Comparison of the impact of the two categories of evidence on sentence by percentage.

In Table 3; Figure 3 below, the data is presented in the form of percentages of the total number of cases in each category. A comparison between Category 1 and Category 2 cases shows the similarity between the proportion of cases where neuroscience tended to aggravate the sentence, mitigate the sentence, and both aggravate and mitigate the sentence.

**Table 3:** Comparison of the impact of two categories of evidence on sentence by percentage.

Description	Category 1 At least one “imaging” evidence	Category 2 At least one “imaging” and/or “non-imaging” evidence (all cases)
Only aggravating	0%	0.3%
Only mitigating	35.9%	37.6%
Both aggravating and mitigating	4.7%	5.3%
Does not contribute to the sentence or unclear	59.4%	56.7%
Contributed to sentence	40.62%	43.23%
Total cases	64	340

Overall, in more than half of the cases in both categories, neuroscience did not contribute to the sentencing decision. This suggests that when neuroscience is submitted to the court, it is more likely that neuroscience does not contribute to the sentence. From the cases where neuroscience contributed to the sentence (around 40% in both categories), the majority of those cases showed that neuroscientific evidence supported a more lenient sentence (in more than 85% in both categories). There is only one case (which is in Category 2) where neuroscientific evidence tended towards a longer sentence and did not simultaneously support a shorter sentence (see *Ngati* above). In around 5% of cases in both categories, neuroscience tended towards both mitigating and aggravating the sentence.

### 5.1. Non-imaging evidence

**Figure 4:**
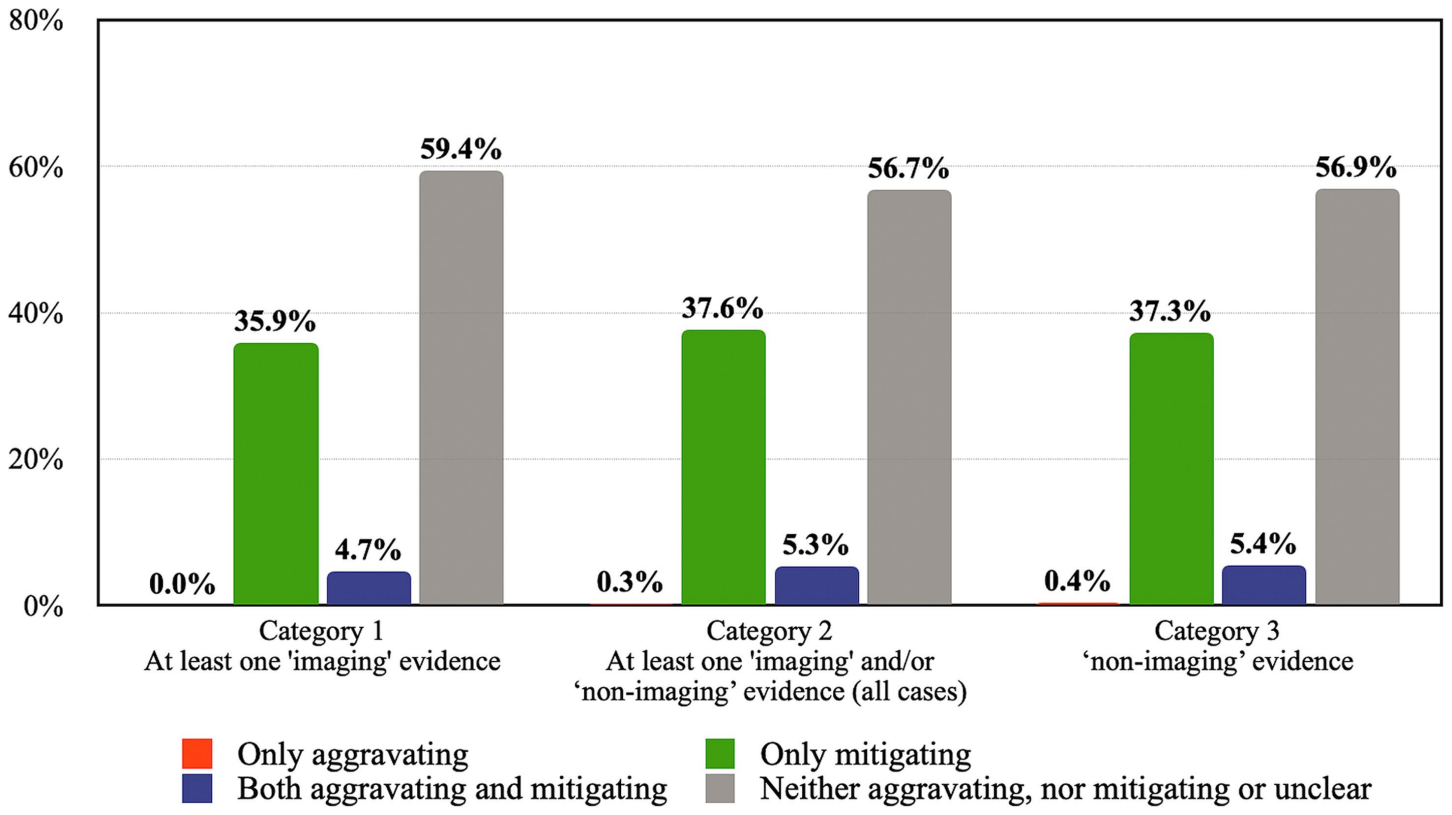
Comparison of the impact of three categories of evidence on sentence by percentage.

While both categories of cases have similar results, it may still be relevant to consider whether cases that do not contain imaging evidence have a different outcome. Accordingly, as shown in Table 4, a third category was created consisting of 276 cases which only involve non-imaging evidence. As seen in Figure 4, a comparison between Category 3 and the other two categories suggests similar outcomes.^22^

**Table 4:** Impact of Category 3 evidence on the sentence.

Description	Category 3 “non-imaging” evidence
Only aggravating	1
Only mitigating	103
Both aggravating and mitigating	15
Does not contribute to the sentence or unclear	157
Contributed to sentence	119
Total cases	276

### 5.2. Neuroscience, a double-edged sword

According to the double-edged sword claim, when neuroscientific evidence is adduced in a case, it may result in either a more lenient sentence in some instances but a harsher punishment in others. For example, neuroscientific evidence may be used to support an argument that the offender has a lower capacity to understand the wrongfulness of their act and consequently mitigate the offender’s culpability. It may be also used to support a harsher punishment if the evidence demonstrates that the offender poses a potential future risk to the community (Jones and Shen, 2012, 362).

As the results of this study suggest, neuroscience appears to support a more lenient sentence substantially more frequently and the theoretical claim that neuroscience acts as a double-edged sword does not appear to be consistent with the use of neuroscience in practise — regardless of whether a broad or narrow definition of neuroscience is used (eg, where cases contain at least one brain-imaging evidence versus cases that contain any type of neuroscientific evidence). Reaching a similar conclusion, American legal scholar Deborah Denno concluded that the double-edged sword claim is a myth and neuroscientific evidence is rarely used to support an argument that the offender will be a potential societal risk. Instead, it is usually introduced with the purpose of mitigating the sentence (Denno, 2015). In the study that was conducted in the Netherlands, out of 231 cases (mix of genetic and neuroscientific evidence cases), there were 15 cases where neuroscientific evidence was explicitly discussed with respect to the risk of future offending (de Kogel and Westgeest, 2015).

This conclusion has an important and practical application for defence lawyers who were previously uncertain about whether introducing neuroscientific evidence would risk increasing the punishment, or, conversely, whether not introducing such evidence would lose an opportunity to mitigate the punishment (for similar discussion see, Barth, 2007; Denno, 2015).

This conclusion might be explained by the fact that predicting offenders’ behaviour some years in the future is more challenging than using different neuroscientific tools to explain their past behaviour. The research in areas of ‘neuroprediction’ and ‘AI neuroprediction’ of recidivism, wherein neuroscience and artificial intelligence are used to predict future recidivism, is still in a nascent stage. Some preliminary studies (see Aharoni et al., 2013, 2014; Delfin et al., 2019; Tortora et al., 2020; Allen, 2021) suggest these predictive models may be capable of more accurately assessing the risk of recidivism (but see, Coppola, 2018; Giannini, 2021; Gkotsi, 2021). This line of research might then be reflected in the expert witnesses’ reports, and future courts might find neuroscientific evidence more frequently favouring harsher punishments.

### 5.3. Sentencing factors and the defendant’s strategy

The conclusion in the previous section with respect to the double-edged sword claim would assist the defence’s decision as to whether they should adduce neuroscientific evidence and risk the chance of receiving a harsher sentence, or withhold the evidence and lose their chance of mitigating the sentence. However, from a defence lawyer’s perspective, knowing which specific aggravating and mitigating sentencing factors are often associated with neuroscience, and how frequently they are associated, can be helpful in designing their strategies.

**Figure 5:**
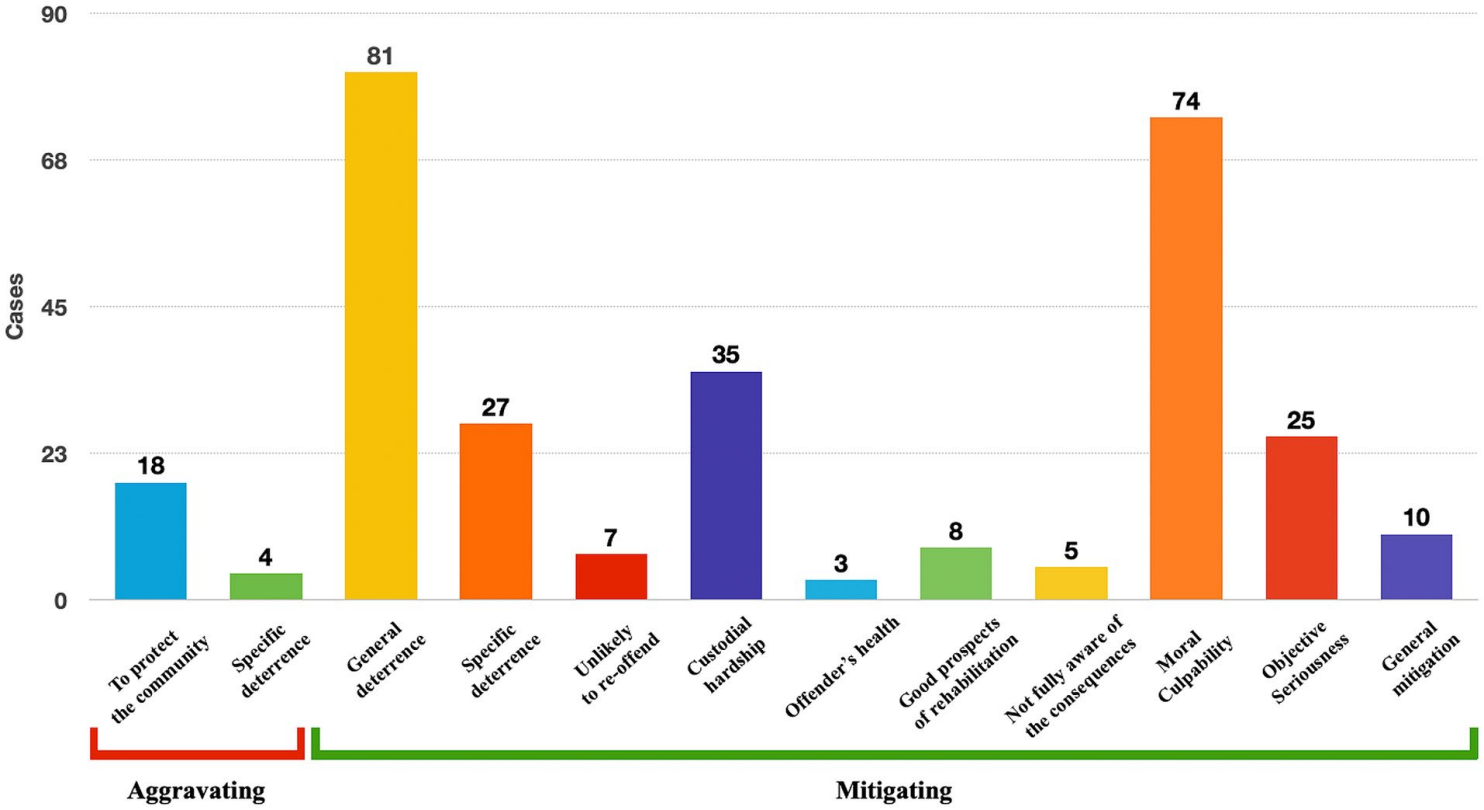
Contribution of neuroscientific evidence to sentencing factors in Category 2, ie, cases with at least one imaging and/or non-imaging evidence (categories are not mutually exclusive).

With respect to Category 2 cases (ie, both imaging and non-imaging evidence), Figure 5 shows several of the sentencing factors that neuroscientific evidence had been found to be associated with and the number of cases associated with each factor. In this study, the sentencing factors were divided into two groups of factors: mitigating and aggravating.^23^ In many cases, neuroscientific evidence was found to be relevant to more than one sentencing factor and therefore, these cases are considered more than once (ie, categories are not mutually exclusive). It should be noted that while there is an overlap between the two concepts of moral culpability and the objective seriousness of the offence, in some cases where there was causal relation between offender’s brain condition (e.g., by reducing offender’s capacity to fully appreciate the rightness or wrongness of their act), the court referred to both concepts explaining that as a result of reduction in offender’s moral culpability, the objective seriousness of the offence was mitigated. And in some cases, the court referred to reduction of either moral culpability or objective seriousness of the offence.

As illustrated in Figure 5, the majority of sentencing factors relevant to neuroscientific evidence were mitigating factors. The two most common mitigating sentencing factors were moral culpability^24^ (74 cases) and general deterrence (81 cases). There were also 10 cases under the ‘general mitigation’ heading which includes cases where it appeared from the judgement (mostly appellate courts) that neuroscientific evidence mitigated the punishment, but it was unclear which sentencing factor was relevant.

Contrastingly, there were only two aggravating factors associated with neuroscience. There were 18 cases concerned with the protection of society and four cases concerned with specific deterrence associated with neuroscientific evidence.

**Figure 6:**
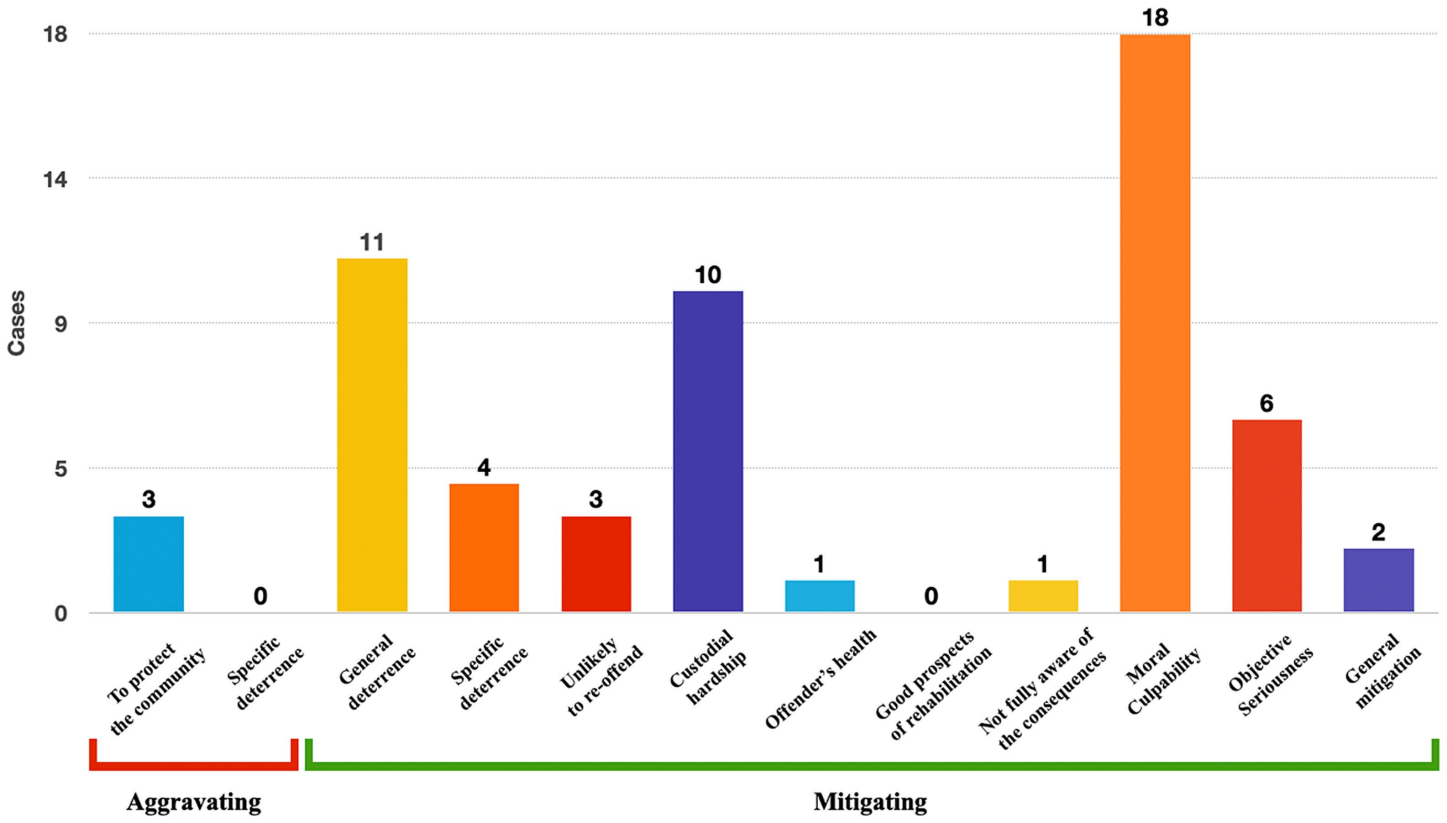
Contribution of neuroscientific evidence to sentencing factors in Category 1, ie, cases with at least one imaging evidence (categories are not mutually exclusive).

With respect to Category 1 cases (imaging evidence), the moral culpability of the offender was the most common mitigating factor associated with neuroscience (18 cases), followed by general deterrence (11 cases) and custodial hardship (10 cases). On the aggravating side, there were three cases associated with protection of the community that may have contributed to a more severe sentence (Figure 6).

These figures suggest that where the defence raises neuroscience evidence to mitigate the punishment, arguing under the factors of moral culpability, general deterrence and custodial hardship would more likely mitigate punishment and should be prioritised in the defence’s strategies.

### 5.4. Unclear cases

A note of caution is due in this analysis. As was discussed in the methodology section, in some cases it was not clear whether neuroscience contributed to sentencing, and hence these cases were coded as ‘unclear’. Some may argue that if in some or all the unclear cases, neuroscience actually tended to aggravate or mitigate the sentence, the conclusion that neuroscience in majority of cases supports a more lenient sentence could be different.

In Category 2, which contained all 340 cases, there are six cases where it was unclear whether neuroscience supported a harsher sentence.^25^ If we assume that in all these six cases neuroscience did in fact support a harsher punishment and that there are no cases where the contribution of neuroscience to mitigating the sentence is unclear, the conclusion made about the general tendency of neuroscience to mitigate the sentence would not be different. In two of the six cases, neuroscience also contributed to mitigating the sentence, and as such the total number of cases that neuroscience contribute to both aggravating and mitigating the sentence would increase from 18 to 20 (that is from 5.3 to 5.9%). In four of the six cases, neuroscience did not contribute to mitigating the sentence, meaning that the total cases where neuroscience only aggravated the sentence would increase from one to five (that is from 0.3 to 1.7%).

These findings are similar for Category 1 (ie, cases with at least one imaging evidence). There were only two cases where it was unclear whether imaging evidence contributed to aggravating the sentence, and in these two cases the brain imaging evidence did not contribute to mitigating the sentence. As such, the total number of cases where imaging evidence only aggravated the sentence would increase from 0 to 2 (that is from 0 to 3.1%).

With these results, it appears that even if in all the unclear instances where it was assumed that neuroscience supported a harsher punishment, this would not affect the overall conclusion that in majority of cases where neuroscience contributed to sentencing, it supported a more lenient sentence.

## 6. Neuroscientific evidence and risks for the defence strategy: two further considerations

While the results of this study suggest that in the majority of cases where neuroscience contributes to sentencing it supports a more lenient sentence, it may still be a risky strategy for defence lawyers to adduce neuroscientific evidence. The two areas that require consideration are as follows:

### 6.1. Cases biased towards mitigation

In the adversarial system, prosecutors have a duty to provide all relevant materials to the court that would assist in the determination of a sentence (see *HT v R* (2019) 269 CLR 403, 425, [59] (Nettle and Edelman JJ), cited in Odgers, 2015).^26^ Therefore, prosecutors have a duty to present neuroscientific evidence at criminal hearings regardless of the influence that it may have on the punishment and whether it may reduce or aggravate the sentence.

Thus, it is possible that defendants may withhold neuroscientific evidence to avoid any potential risk that the evidence could be used to aggravate the punishment. The natural reluctance of defendants to raise neuroscientific evidence in such cases may be a confounding factor in the sample used in this study and may have affected the conclusion reached, being that neuroscientific evidence points towards mitigation in the majority of cases.

To illustrate this issue more clearly, *Sumpton v The Queen* [2016] NSWCCA 162 will be discussed, a case where it appears that the defence had decided not to tender an expert report on an assessment of the offender’s brain damage. Sumpton was convicted of arson and murder for striking the deceased with a statue, stabbing her 24 times and setting a house on fire.

The offender appealed against both conviction and sentence on various grounds. With respect to the sentence appeal, the applicant claimed that the sentencing judge could not take the psychiatrist’s report into consideration as his previous legal representatives had decided not to present this evidence to the court. He claimed that the psychiatrist’s report revealed a high probability of the applicant suffering from alcohol-related brain damage associated with a frontal lobe injury, thereby impairing his judgement. It was also claimed that the brain damage could potentially decrease his tolerance towards the disinhibiting effect of alcohol. Accordingly, the sentencing judge was ‘dealing with an offender with brain damage which may reasonably be supposed to have impacted on the offence committed’ (at [118]). The defence argued that the sentence imposed could have been different if the expert report had been submitted to the court.

The Crown argued against the claim that there had been a miscarriage of justice because the “applicant’s then legal representatives had a number of legitimate concerns arising from other observations made by [the expert witness] in his report, and had made a forensic decision, in accordance with the applicant’s instructions, not to tender the report on sentence” (at [120]). Further, the Crown submitted that as the report was available during the trial court, it could not be considered as fresh evidence and could not be considered for the appeal.

The appellate court noted that the expert witness did not report on whether the diagnosed brain injury was causally associated with the offences committed by the applicant. Rather, the expert explained that there would be a risk of recidivism if the applicant did not cease his alcohol consumption. The Court considered this to be one of the main explanations of why the report had not been tendered in the lower court. The appellate court decided that there was no miscarriage of justice and the appeal was dismissed.

It is unclear how many cases similar to *Sumpton* exist, where the defence lawyer decides to withhold relevant neuroscientific evidence. This makes it difficult to ascertain whether and to what extent this issue may affect the conclusions drawn on the use of neuroscience in sentencing.

That said, in cases where it appeared that neuroscience would simultaneously support a harsher sentence and mitigate the sentence by way of moral culpability, taking the risk of tendering neuroscientific evidence seemed to be a justifiable decision for defendants. As discussed in ‘Sentencing Factors Associated with Neuroscience’, the High Court in *Veen No 2* explained that in cases where mental condition is associated with the countervailing effects of moral culpability and protection of society, the consideration of the protection of society cannot result in a sentence that is more severe if the offender did not have a mental abnormality (at 465, 477, Mason CJ, Brennan, Dawson and Toohey JJ). Further, as discussed in Part Three, the High Court’s conclusion appears to be based on the fact that protection of society is a subjective factor that cannot increase the severity of the sentence beyond the proportionate punishment (465, 472, Mason CJ, Brennan, Dawson and Toohey JJ). Since specific deterrence is also a subjective sentencing factor, none of the two sentencing factors that are potentially associated with neuroscientific evidence can extend the sentence beyond the proportionate punishment. Meanwhile, in cases where a brain condition is causally associated with the offence, moral culpability influences the objective seriousness of an offence and the proportionate sentence and, therefore, carries more weight in sentencing compared to subjective factors.

### 6.2. Legislation and the risk of a longer detention

There are two Acts that lawyers may need to consider before adducing neuroscientific evidence based on the circumstances of the case: section 61(1) of the *Sentencing Act* and the *Crimes (High Risk Offenders) Act 2006* (NSW).

According to section 61(1) of the *Sentencing Act*, the court is to impose mandatory life sentences (ie imprisonment for the term of offender’s natural life) in murder crimes:

if the court is satisfied that the level of culpability in the commission of the offence is so extreme that the community interest in retribution, punishment, community protection and deterrence can only be met through the imposition of that sentence.^27^

Section 61(3) also explains that subjective factors may tend towards a sentence lower than that of life imprisonment.^28^ On the other hand, if the offender’s level of culpability (ie associated with the objective seriousness of the offence) makes the offence one that is so extreme, the court may impose the sentence regardless of the subjective circumstances of crime (eg, the offender’s prospects of rehabilitation) (see *R v Ngo (No 3)* (2001) 125 A Crim R 495, 503 [42]).^29^ As such, because one of the components of section 61(1) of the Act is the protection of society, in cases of murder offences, where neuroscientific evidence supports a high risk of recidivism, the defendant may need to consider whether it is worth taking the risk of adducing neuroscientific evidence to receive a more lenient sentence, or withhold the evidence so as to avoid an order of life sentence under the Act.

Other than section 61 of the *Sentencing Act*, there is another legislative instrument that creates risks when tendering neuroscientific evidence to the court. Section 3 of the *Crimes (High Risk Offenders) Act 2006* (NSW) outlines the aims of the legislation, to protect the community from sex and violent offenders by preventing the commission of further serious offences after offenders serve their sentence, and to encourage rehabilitation of these offenders.^30^ The Act provides that for high-risk sex and violent offenders, the Supreme Court, prior to the end of their sentence, may impose post-sentence continuing (preventive) detention which obliges the offender to remain in custody after serving their sentence. The Supreme Court may also impose ongoing supervision, ie restricting the offender’s liberty in ways such as through requiring the offender to make periodic reports to a corrective services officer and joining rehabilitation programmes.^31^ These orders are imposed where ‘the Supreme Court is satisfied to a high degree of probability that the offender poses an unacceptable risk of committing another serious offence if not kept’ under supervision or under a detention order (*Crimes (High Risk Offenders) Act 2006* (NSW) ss 5B-5C). One important condition of this Act is that such offences must be serious. For example, the Act defines serious violent offences as involving:

engaging in conduct that causes the death of another person or grievous bodily harm to another person, with the intention of causing, or while being reckless as to causing, the death of another person or grievous or actual bodily harm to another person (Crimes (High Risk Offenders) Act 2006 (NSW) s 5A(1)(a)).

An example that illustrates where the court may potentially impose an order under this Act is *R v Ray* [2013] NSWSC 767, where the offender was convicted of murder. Although the offender did not intend to murder his victim, he ‘inflict[ed] really serious bodily harm’ during a savage assault (at [2]). The offender had an extended criminal record including violence against women.

A neuropsychologist, following a review of the offender’s history, and neuropsychological and psychometric testing concluded that the offender’s

behaviour and type of cognitive deficits are consistent with those seen in patients with damage to … [part of the] … pre-frontal cortex of the brain. This area of the brain plays a key role in impulse control and in regulation and maintenance of set and ongoing behaviour. Damage to this region can result in disinhibition and impulsivity with such associated behaviour problems as aggressive outbursts and sexual promiscuity. It is reported many patients with … [such brain] … damage develop problems with social conduct, as well as defects in planning judgement and decision making (at [69]).

As there was no brain imaging evidence to confirm the results of the medical examinations, the neuropsychologist expressed some reservations about the interpretation of the offender’s brain injury.

The Court reduced the weight given to considerations of the offender’s moral culpability and general deterrence due to his brain injury. However, because of the offender’s criminal history, the Court considered that reduction to a minimal extent.

As there was no remedy or cure for the offender’s brain damage, the expert witness suggested that participating in intensive rehabilitation programmes may be helpful. The Court noted that unless the offender was successfully involved in appropriate rehabilitation programmes during his sentence, he would continue to impose a danger to society, particularly to women. The Court concluded that protection of society was a significant consideration in the case, but ‘[t]hat does not mean that his sentence can be extended merely to protect society; the law is clear that a sentence must remain proportionate to the offence. But with that constraint in mind, protection of the community is a significant consideration in this case’ (at [76]).

Due to the nature of the offence, the judge commented about the application of the *Crimes (High Risk Offenders) Act 2006* (NSW), warning the offender that:

this means that the State may make an application at the end of his sentence for him to be made the subject of a continuing detention order or an extended supervision order for up to 5 years and that the State may continue to make applications for further such orders … It would be in his interests to engage with rehabilitation programmes whilst serving his sentence. If he does not, there is the potential for him to remain in custody for the rest of his life (at [80]).

It is of note that during sampling the cases in this study, there were few judgements that refer to the *Crimes (High Risk Offenders) Act 2006* (NSW). Where it did apply, in most cases the decision on the application of this Act was inconclusive. In some cases, either the neuroscientific examination did not suggest the existence of a brain abnormality,^32^ or the court requested further mental examinations (*New South Wales v Thomas* [2009] NSWSC 1410; *New South Wales v Hill* [2014] NSWSC 1803) rather than making a conclusive decision (See *New South Wales v Scott* [2013] NSWSC 1834). These judgements are not included in the 331 cases evaluated in this study. This is mainly because the orders made at the end of the offender’s sentence are not part of the sentencing process and the majority of court decisions on the application of this Act was inconclusive.

The influence of neuroscientific evidence with respect to the application of this Act requires further examination in the future. This is because before 2014, the application of this Act was limited to cases involving sex offenders. However, since the amendments in 2014, the Act now also applies to high-risk violent offenders.^33^ As this study is limited to judgements published prior to the end of 2016, it is unclear the extent to which this Act may create issues relating to neuroscientific evidence.

## 7. Conclusion

This study investigated how neuroscientific evidence has been considered in sentencing decisions in NSW criminal courts. The results of this study indicate that neuroscience contributed to sentencing in less than half of the cases examined. Of these cases that contributed to sentencing, there is a considerably higher number of instances where neuroscientific evidence supported the mitigation, rather than the aggravation of the sentence or both, and this conclusion does not differ based on the different categories of evidence (ie, categories of the narrow or broad definition of neuroscientific evidence). These findings also suggest that the use of neuroscientific evidence in NSW criminal courts is inconsistent with the double-edged sword claim.

While the results of this study suggest that neuroscience evidence often supported a more lenient sentence, the introduction of such evidence may pose risks for the post-sentencing phase. In cases where there is a high risk of recidivism (eg, due to untreated brain damage), the offender may be the subject to a continuing detention order, an extended supervision order (*Crimes (High Risk Offenders) Act 2006*) or, in murder cases, life imprisonment might be an option (s 61(1) of the *Sentencing Act*).

It is important to note that this study is subject to a range of methodological limitations and the results should be interpreted with a degree of caution. Criminal procedure is a rather complicated matter and there are potentially hidden or confounding factors that may not have been considered in this study. Given these limitations, future studies are recommended to further investigate this area using other methods, such as interviewing judges to better understand their decision-making process when considering neuroscience and other forms of evidence in sentencing and how they consider experts’ qualifications in reporting on different forms of neuroscience evidence. Similarly, interviewing prosecutors would help to identify what factors may encourage them to introduce neuroscientific evidence to point out the risk of recidivism and whether more research in areas such as ‘neuroprediction’ would change their strategies.

By evaluating the use of neuroscience in sentencing, this study has provided only a narrow perspective on the vast intersection of law and neuroscience in courts. In the process of examining criminal judgements, other important neurolaw themes were identified that merit future empirical research. These areas include criminal responsibility and young offenders’ brain development, the fitness of defendants with brain impairment to stand trial, and the insanity defence (i.e., defence of mental health impairment or cognitive impairment).

This study’s findings, while directly applicable only to the specific Australian jurisdiction examined, establish a foundation for cross-comparative analysis with other jurisdictions such as the United States, Canada, Slovenia, the Netherlands, and England and Wales, where similar empirical research has been conducted. Consequently, these studies could collectively contribute to a more profound understanding and create a holistic representation of the application of neuroscientific evidence worldwide.

It is hoped that this study will contribute to shifting the discourse on law and neuroscience from theory to practise in courts, and will stimulate further discussion and research on the use of neuroscience both in Australian courts and other jurisdictions.

## Data availability statement

The raw data supporting the conclusions of this article will be made available by the authors, without undue reservation.

## Author contributions

The author confirms being the sole contributor of this work and has approved it for publication.

## Funding

Part of this research was conducted during my PhD at UNSW, Australia, where I was awarded the University International Postgraduate Award (UIPA).

This research was supported by the University of Wollongong, Australia.

## Conflict of interest

The author declares that the research was conducted in the absence of any commercial or financial relationships that could be construed as a potential conflict of interest.

## Publisher’s note

All claims expressed in this article are solely those of the authors and do not necessarily represent those of their affiliated organizations, or those of the publisher, the editors and the reviewers. Any product that may be evaluated in this article, or claim that may be made by its manufacturer, is not guaranteed or endorsed by the publisher.

## Footnotes

^1^Similarly, Owen D. Jones and Francis X. Shen summarise the double-edged sword claim in this way, ‘a brain *too* broken may be simply too dangerous to have at large, even *if* it is somehow less culpable’ (emphasis in original): Jones and Shen (2012), 362. Also see Barth (2007), Snead (2007), and Farahany and Coleman Jr (2009).

^2^The question of whether neuroscience is useful, and whether it is overly persuasive are matters beyond the scope of this article.

^3^Unlike other imaging techniques, EEG and MEG do not generate an actual image of the brain – rather they produce brainwave forms of brain activity. Nonetheless, these techniques are included with other imaging techniques in many neurolaw studies, possibly because they are non-invasive approaches that can extract information about brain function. See, Moriarty (2008) and Greely and Wagner (2011).

^4^In this regard, Paul Catley and Lisa Claydon explain that ‘tests developed in whole or in part on the basis of brain scan findings, but which do not themselves involve scans could arguably fall either side of the line. Artificial distinctions are difficult’ (Catley and Claydon, 2015, 514).

^5^Some neurolaw studies also have defined both brain imaging and non-brain imaging evidence as neuroscientific evidence, see Denno (2015) and Farahany (2016).

^6^Caselaw provides decisions specifically for Courts of NSW and Tribunals administered by the NSW Department of Justice. Caselaw’s collection of cases is also confined to specific courts and periods including: cases issued after 1999 from the Court of Criminal Appeal; cases issued after 2005 from the District Court (incomplete collection); cases issued after 2002 from the Local Court (incomplete collection); cases issued after 1999 from the Supreme Court: ﻿https://www.caselaw.nsw.gov.au/about.

^7^Australasian Legal Information Institute (AustLII) claims to be the most popular online free-access resource for Australian legal information, and has over 700,000 daily visits: ﻿http://www.austlii.edu.au. AustLII’s collection of judgements is confined to specific time periods and courts. For example, it provides judgements from the Supreme Court of New South Wales from 1995 to present (three cases are available before 1995) and the collection is limited to the selected decisions of the Court: ﻿http://www7.austlii.edu.au/cgi-bin/viewdb/au/cases/nsw/NSWSC. The selected decisions of the Supreme Court of New South Wales and Court of Criminal Appeal include those from 1999 to the present (five cases are available before 1999): ﻿http://www6.austlii.edu.au/cgi-bin/viewdb/au/cases/nsw/NSWCCA. AustLII also provides selected decisions from the District Court of New South Wales from 2005 to the present. According to AustLII, ‘[b]efore 2004, these decisions were exclusively appeals made by pharmacists against the disciplinary findings and/or orders made by the Pharmacy Board of New South Wales. The headnote to each appeal decision has a link back to the relevant Pharmacy Board decision’. Only 15 judgements of this type from before 2005 are available: ﻿http://www.austlii.edu.au/au/cases/nsw/NSWDC/.

^8^Some of the cases extracted from Australian Neurolaw Database have not been published yet and are not available to the public.

^9^All of which are associated with non-imaging evidence.

^10^See footnotes 7 and 8 for AustLII and CaseLaw collection of different courts judgements.

^11^The case collection and analysis of judgment are partly derived from my PhD project at the University of New South Wales, Australia, 2015–2019.

^12^Also, in *Muldrock v The Queen* (2011) 244 CLR 120, 140 [58], the High Court of Australia held that ‘punishment, in the sense of retribution, and denunciation did not require significant emphasis in light of the appellant’s limited moral culpability for his offence’. *Bugmy v The Queen* (2013) 249 CLR 571, 594 [40]: “The circumstance that an offender has been raised in a community surrounded by alcohol abuse and violence may mitigate the sentence because his or her moral culpability is likely to be less than the culpability of an offender whose formative years have not been marred in that way.”

^13^*Crimes (Sentencing Procedure) Act* 1999 (NSW) (“*Sentencing Act*”) ss 3A(d), 21A(3)(h).

^14^*Sentencing Act* s 3A(c). See also *R v Z* (2006) 167 A Crim R 436, 448 [50], where Beazley JA discusses how different sentencing factors, including protection of society, are associated with the mental condition of the offender.

^15^*Sentencing Act* s 21A(3)(g).

^16^*Sentencing Act* s 3A(a). Also see “just deserts” approach: Odgers (2015), 73.

^17^See also Brown et al. (2020), 1,339; *Kennedy v T* (2008) 181 A Crim R 185, [39]. Sperling J in *R v Hemsley* [2004] NSWCCA 228, [33] discussed that ‘where mental illness contributes to the commission of the offence in a material way, the offender’s moral culpability may be reduced; there may not then be the same call for denunciation and the punishment warranted may accordingly be reduced’. Also Hodgson JA noted that ‘[i]n my understanding, considerations of retribution direct attention to what the offender deserves; and in my opinion, where emotional immaturity or a young person’s less-than-fully-developed capacity to control impulsive behaviour contributes to the offending, this may be seen as mitigating culpability and thus as reducing what is suggested by considerations of retribution’: *BP v The Queen* (2010) 201 A Crim R 379, 381 [4] (Rothman J agreeing; Johnson J dissenting in the principal judgement) (citations omitted).

^18^It should be noted that different factors can affect moral culpability of the offender, however in this study where I refer to reduced significance of moral culpability, it is in situations that offender’s mental condition, e.g., by impairing their judgement, is causally connected to the offence and that affected the consideration of objective seriousness of the offence.

^19^See also *Kennedy, James Anthony v The Queen* (2008) 181 A Crim R 185, 195 [38] (Beazley JA).

^20^Similarly, rehabilitation as a subjective factor cannot justify a longer sentence above what is proportional to the objective seriousness of the offence *R v Royal* (2003) 40 MVR 563, 564 (Wood CJ at CL and Howie J), cited in ﻿https://www.judcom.nsw.gov.au/publications/benchbks/sentencing/index.html.

^21^For a similar discussion on this case see, Alimardani and Chin (2019).

^22^The results in Figure 3 also suggests an inconsistency with the claim that brain imaging evidence may have a more persuasive outcome in comparison to non-imaging evidence. This conclusion, however, is subject to the limitations of this study. Further, an evaluation of this claim requires detailed analysis that I will do in another study.

^23^As previously discussed under ‘Sentencing Factors Associated with Neuroscience’, some of the sentencing factors listed in these Figures are not mitigating or aggravating in and of themselves. For example, in cases where the court attached greater weight to the significance of specific deterrence, I considered it as an aggravating factor. And in cases where specific deterrence received a lower weight, I considered it as a mitigating factor.

^24^As previously discussed under ‘Sentencing Factors Associated with Neuroscience’, these are the instances where the moral culpability of the offender was reduced due to the fact that the brain condition was causally connected to the offending by way of, e.g., reducing offender’s capacity to reason.

^25^There are two aggravating sentencing factors that neuroscience may be relevant. Protection of society and specific deterrence. In six cases, it was unclear whether neuroscience contributed to the consideration of protection of society, and in three cases it was unclear whether neuroscience was associated with attaching more weight to specific deterrence. If it was suggested that one of these two factors contributed to the sentence, the case was considered as an instance where neuroscience tended towards a harsher punishment, regardless of whether the other factor contributed to the sentence or whether the contribution of the other factor was unclear. Accordingly, the total number of cases where it was unclear that neuroscience contributed to a harsher sentence is six.

^26^Also see, ﻿https://www.judcom.nsw.gov.au/publications/benchbks/sentencing/index.html.

^27^A life sentence may also be imposed for serious heroin or cocaine trafficking offences in cases where additional criteria are met, including where the offence involves “a high degree of planning and organisation” under section 61(2) of the *Sentencing Act*. However, since drug trafficking offences are not relevant to the context of this section, this was not included in the discussion.

^28^Section 61(3) of the *Sentencing Act* notes that, ‘Nothing in subsection (1) affects section 21(1)’. Section 21 of this Act, under the general power to reduce penalties, provides that ‘(1) If by any provision of an Act an offender is made liable to imprisonment for life, a court may nevertheless impose a sentence of imprisonment for a specified term. There are some arguments around the applicability of section 21(1) to permit discretion in mitigating the punishment despite section 61(1) requiring mandatory life imprisonment where the criteria set in that section are met. It appears this issue is addressed in favour of s 21(1): ﻿https://www.judcom.nsw.gov.au/publications/benchbks/sentencing/index.html.

^29^﻿https://www.judcom.nsw.gov.au/publications/benchbks/sentencing/index.html. Moral culpability may be reduced because of the existence of a brain impairment. However, it may instead be increased because of aggravating factors, such as where an offender has committed sexual assault in the company of others. In some certain circumstances, the court may find that the offence falls under the most extreme level of moral culpability, for instance where murder and mutilation of a child is committed by a relative. See *R v Whyte* (2002) 55 NSWLR 252, 284 [205], 287–8 [231], cited in ﻿https://www.judcom.nsw.gov.au/publications/benchbks/sentencing/index.html.

^30^There are some criticisms of this Act. For instance, some argue that the Act abrogates principles of human rights and law. See, Bartels et al. (2019). For more information about the application of this Act in NSW see, Tulich (2015).

^31^See *Crimes (High Risk Offenders) Act 2006* (NSW) ss 11, 13B, 18. This order can be interim or continuing: *Crimes (High Risk Offenders) Act 2006* (NSW) ss 10A, 18A.

^32^The court made an interim order. See sections 10A and 18A of the *Crimes (High Risk Offenders) Act 2006* (NSW).

^33^In 2013, the Act’s title was changed from the *Crimes (Serious Sex Offenders) Act 2006* (NSW) to the *Crimes (High Risk Offenders) Act 2006* (NSW), along with further amendments.
